# Adiponectin improves coronary no-reflow injury by protecting the endothelium in rats with type 2 diabetes mellitus

**DOI:** 10.1042/BSR20170282

**Published:** 2017-07-27

**Authors:** Xue Han, Ye Wu, Xin Liu, Lu Ma, Tingting Lv, Qi Sun, Wenli Xu, Suli Zhang, Ke Wang, Wen Wang, Xinliang Ma, Huirong Liu

**Affiliations:** 1 Department of Physiology and Pathophysiology, School of Basic Medical Sciences, Capital Medical University, Beijing 100069, PR China; 2 Beijing Key Laboratory of Metabolic Disorders Related Cardiovascular Disease, Capital Medical University, Beijing 100069, PR China; 3 Institute of Molecular Medicine, Peking University, Beijing 100871, PR China; 4 Department of Emergency Medicine, Thomas Jefferson University, Philadelphia, PA 19107, USA

**Keywords:** adiponectin, coronary no reflow, endothelial cell, microcirculation, type 2 diabetes mellitus

## Abstract

To determine the effect of adiponectin (APN) on the coronary no-reflow (NR) injury in rats with Type 2 diabetes mellitus (T2DM), 80 male Sprague–Dawley rats were fed with a high-sugar–high-fat diet to build a T2DM model. Rats received vehicle or APN in the last week and then were subjected to myocardial ischemia reperfusion (MI/R) injury. Endothelium-dependent vasorelaxation of the thoracic aorta was significantly decreased and serum levels of endothelin-1 (ET-1), intercellular cell adhesion molecule-1 (ICAM-1) and vascular cell adhesion molecule-1 (VCAM-1) were noticably increased in T2DM rats compared with rats without T2DM. Serum APN was positively correlated with the endothelium-dependent vasorelaxation, but negatively correlated with the serum level of ET-1. Treatment with APN improved T2DM-induced endothelium-dependent vasorelaxation, recovered cardiac function, and decreased both NR size and the levels of ET-1, ICAM-1 and VCAM-1. Hypoadiponectinemia was associated with the aggravation of coronary NR in T2DM rats. APN could alleviate coronary NR injury in T2DM rats by protecting the endothelium and improving microcirculation.

## Introduction

Acute coronary syndromes (ACS), especially acute myocardial infarction (AMI), are very dangerous and have a high mortality. Previously, continuous and effective myocardial reperfusion therapy was proven to reduce significantly the myocardial infarction size, decrease the mortality and improve the prognosis of patients. In clinical practice, percutaneous coronary intervention (PCI) has become the most important method for cardiac revascularization [1]. However, approximately 10–30% of patients with AMI experience coronary no-reflow (NR), after epicardial coronary stenosis or occlusion is relieved by vascular recanalization with PCI. Coronary NR [2], may lead to severe hemodynamic disorders, cause poor left ventricular remodeling and early post-infarction heart failure, and affect the prognosis of patients, or even increase mortality during hospitalization [3]. A variety of treatment strategies have been used to prevent or treat the phenomenon of NR, however, a complete and effective strategy has not yet been established, since different patients may have different pathogeneses. Therefore, it is important to investigate the mechanism of NR, so as to improve the therapeutic effectiveness for ACS patients, and reduce and avoid the adverse cardiac events caused by NR.

Patients with type 2 diabetes mellitus (T2DM) have a higher incidence of ACS than the normal population. Moreover, they usually have a larger infarction size and more severe damage than non-diabetes mellitus (DM) patients, which might lead to a poor therapeutic effects after reperfusion, and increase significantly the risk of coronary NR and mortality [4]. Therefore, it is of great importance to prevent increased NR risk in patients with T2DM so as to improve cardiac function and decrease mortality.

Adiponectin (APN) is a fatty cytokine secreted by adipocytes, which is currently regarded as having three kinds of biological effect: (1) insulin sensitizing (insulin metabolism adjusting), (2) anti-inflammatory (vascular protective) and (3) anti-ischemic injury (cardioprotective). Interestingly, the plasma level of APN is correlated negatively with the risk of ACS. A large number of epidemiological studies have found that most of patients with T2DM have hypoadiponectinemia [5]. Moreover, the risk of cardiovascular diseases will increase correspondingly with the decrease in APN levels in patients with DM or adiposis [6]. At present, it is generally believed that systemic APN dysfunction causes the progression of coronary heart diseases and affects the outcome of these diseases in patients with T2DM. Furthermore, giving exogenous APN alleviates myocardial ischemia/reperfusion (MI/R) injury significantly [7]. Therefore, whether decreased APN levels could aggravate NR damage following MI/R in T2DM patients is worth further investigation.

In this study, based on an adiposis-induced T2DM pathological model simulated by continuously feeding a high-sugar–high-fat diet, an NR model was established by MI/R operation to observe whether low APN levels are related to the aggravation of coronary NR in T2DM. In addition, the potential for APN to alleviate coronary NR injury by protecting vascular endothelium and improving myocardial microcirculation disorders was also investigated by supplementing exogenous globular domain of APN (gAd).

## Materials and Methods

### Animals and dietary protocol

This study was performed in accordance with the Guiding Principles in the Use and Care of Animals, published by the National Institutes of Health (NIH Publication No. 85—23, Revised 1996) and approved by the Institutional Animal Care and Use Committee of Capital Medical University.

Eighty healthy male Sprague–Dawley (SD) rats weighing 110.0±10.0g were divided randomly into two groups. The control group (*n*=24) was given a normal diet. The high-sugar–high-fat diet group (*n*=56) was given a diet containing 20% sucrose, 10% triglyceride, 2.5% cholesterol and 0.5% sodium cholate (Huafukang Bioscience Company, Beijing, China) for 32 weeks. Blood was drawn from the tail of each rat. Body weight, fasting blood glucose, fasting serum insulin, the homeostasis model assessment of insulin resistance (HOMA-IR) and serum APN levels were monitored regularly. Starting at 8 weeks into the 32-week diet, intraperitoneal injections of glucose tolerance test (IPGTT) were performed every 4 weeks. An abnormal IPGTT was selected as the indicator of a successful model of T2DM. The T2DM rats were randomly assigned to receive either vehicle (0.9% sodium chloride injection, 0.5 ml/day, tail vein injection, *n*=11) or gAd (20 μg/kg/day, tail vein injection, *n*=12) (Biovision, USA) for the final week.

### Fasting blood glucose test

After an 8 h fast, the glucose levels in the blood from the tail veins were measured using a glucose meter (OneTouch SureStep Blood Glucose Meter, Johnson & Johnson Medical ltd., USA). Fasting blood glucose (FBG) was monitored every 4 weeks.

### Fasting serum insulin test

After an 8 h fast, the serum insulin levels in the blood from the tail veins were measured with an [^125^I]Insulin Radioimmunoassay Kit (Beijing Northern Biological Technology Research Institute, China). Fasting serum insulin (FINS) was monitored every 4 weeks.

### HOMA-IR analysis

The HOMA-IR was calculated according to the following formula:

$$\text{HOMA-IR}=\frac{\text{FBG}\times \text{FINS}}{22.5}$$

where FBG and FINS are measured in mmol/l and IU/ml respectively.

### IPGTT examination

After an 8 h fast, a 2 g/kg 50% glucose solution was injected intraperitoneally, and then the glucose levels in the blood from the tail veins were measured at 0, 30, 60 and 120 min using a glucose meter. Starting at week 8, IPGTT was examined every 4 weeks.

### Animal experimental protocol

At the end of the 32 weeks experimental period, rats were anesthetized with 10% chloral hydrate solution (0.3 ml/100 g). Myocardial ischemia was developed by exteriorizing the heart *via* a left thoracic incision and occluding the left anterior descending (LAD) coronary artery with a 6-0 silk slipknot. After 90 min of ischemia, the slipknot was released and the myocardium was reperfused for 12 h. The sham-operated control rats underwent the same surgical procedures except that the LAD was not occluded.

Rats were assigned randomly to one of four groups: (1) Sham + normal diet group: LAD without occlusion, total time course 13.5 h; (2) MI/R + normal diet group: LAD with reversible occlusion, 90 min ischemia followed by 12-h reperfusion; (3) MI/R + Vehicle + T2DM group: LAD with reversible occlusion, 90 min ischemia followed by 12-h reperfusion; (4) MI/R + gAd + T2DM group: LAD with reversible occlusion, 90 min ischemia followed by 12-h reperfusion.

### Fasting serum APN assay

After an 8 h fast, the serum APN levels in the blood from the tail veins were measured with Rat Total Adiponectin/Acrp30 Quantikine ELISA Kit (R&D Systems, Inc., USA) in accordance with the manufacturers’ instructions. Fasting serum APN was monitored every 2 weeks.

### Determination of myocardial function

At the end of the 12-h reperfusion period, left ventricular (LV) function was continuously monitored via catheter pressure transducer inserted into the LV via left carotid artery [8]. Left ventricular end diastolic pressure (LVEDP), left ventricular systolic pressure (LVSP), and maximal rate of rise/decrease of left ventricular pressure (± dp/dt_max_) were derived by BL-410 biological signal recording and analysis system.

### Determination of NR size

The rats' coronary NR ranges and ischemia areas were observed by Thioflavin-S (Sigma-Aldrich, USA), Evan's blue (Sigma-Aldrich, USA) and fluorescent microsphere (1–10 μm Molecular probes, Thermo Fisher Scientific Inc., USA) staining as in previous studies [9,10]. At the end of the 12-h reperfusion period, 1 ml/kg of a 4% solution of Thioflavin-S [9] was injected into the heart to define the region of NR. Thioflavin-S shows bright blue–green fluorescence in 420-nm UV light, staining the endothelium, serving as a marker of perfusion. After MI/R, Thioflavin-S was unable to flow into NR area due to microvascular obstruction, therefore, Thioflavin-S was used to observe zones of NR. After 1 min, 1 ml/kg of a 2% solution of red fluorescent microsphere [10] was injected into the heart to observe the NR. Red fluorescent microspheres show bright pink fluorescence in 420 nm ultraviolet (UV) light. The diameter of fluorescent microsphere was similar to erythrocyte, the fluorescent microsphere was unable to enter NR area due to microvascular obstruction. Therefore, the distribution of red fluorescence reflects cardiac perfusion in ischemia part. After 1 min, LAD was re-ligated, 2 ml of 2% Evan's blue was injected into the heart via the left auricle, then the rat was killed and the heart obtained. The left and right auricle, right ventricle and remaining vessels were cut off, residual blood was washed in ice heparin saline. The heart was sliced transversely into 6–8 pieces and photographed. The slices were photographed under 420 nm UV light to identify the area of **NR (ANR)** and under standard lighting to identify the **area at risk (AAR)**. The ANR shows no fluorescence, the area of reflow shows fluorescence, the non-ischemia area stains blue, and AAR does not stain blue. The area described above was measured with Image-Pro Plus 6.0 software (Media Cybernetics, Inc., USA), AAR/LV calculates ligation area, ANR/AAR reflects coronary NR range. The higher ratio represents more severe injury.

### Serum ET-1, ICAM-1 and VCAM-1 assay

Blood from the rats was collected at the end of 32 weeks and reperfusion for ET-1, ICAM-1 and VCAM-1 quantitation was carried out to confirm endothelium injury and microcirculation disturbance. The serum was analyzed spectrophotometrically (SoftMax Pro Software; Molecular Devices, Sunnyvale, USA) for ET-1, ICAM-1 and VCAM-1 concentration with rat ET-1, ICAM-1 and VCAM-1 ELISA kits (Wuhan ColorfulGene Biological Technology Co., LTD, China) in accordance with the manufacturer's instructions [11].

### Thoracic aorta preparations and measurement of thoracic aorta vasorelaxation

When the rats were anesthetized, thoracic aortas were removed quickly and placed in ice-cold Hepes-bufferred, glucose-supplemented isotonic salt solution (mmol/l: NaCl 144.0, KCl 5.8, MgCl_2_· 6H_2_O 1.2, CaCl_2_ 2.5, glucose 11.0, Hepes 5.0, pH 7.4). The surrounding tissue was cleaned and the aortas were cut into rings of 2 mm in length, and thoracic aorta rings were attached to two wires (200 μm) connected to an isometric force transducer ((DMT610M, Danish Myo Technology). The rings were suspended in organ bath chambers containing oxygenated (95% O_2_, 5% CO_2_) Hepes buffer and maintained at 37°C. The artery rings were stretched step by step to an optimal resting tension of 100 mmHg, which was maintained throughout the experiment. The rings were equilibrated for 2 h before vasorelaxation measurements were taken, and the Hepes was replaced every 20 min. After the equilibration period, the artery segments were exposed to Hepes buffer containing 60 mM potassium (mM: NaCl 89.8, KCl 60, MgCl_2_· 6H_2_O 1.2, CaCl_2_ 2.5, Glucose 11.0, Hepes 5, pH 7.40) until reproducible contractile responses were obtained. After washing with Hepes buffer, rings of thoracic aorta were precontracted with norepinephrine (10^–5^ mol/l). Once a stable contraction was achieved, increasing concentrations of vasodilators (10^–9^–10^–5^ mol/L) were added to the chamber to obtain cumulative concentration–response curves. Endothelium-dependent dilation was measured with acetylcholine (ACh), and endothelium-independent dilation was measured with sodium nitroprusside (SNP).

### Detection of APN protein by Western blot

After Thioflavin-S, Evan's blue and fluorescent microsphere staining, the heart was quickly excised. The myocardium from right ventricle, the NR area and reflow area of the ischemia area in the same rat was harvested for APN protein detection using Western blot. Briefly, the tissue proteins were separated by SDS–PAGE and transferred to nitrocellulose membranes. After being blocked with 3% bovine serum albumin, the immunoblots were probed with the primary antibody (anti-APN and anti-β-actin) overnight at 4°C. The membranes were incubated with goat anti-rabbit IgG H&L (HRP) (1:3,000, Abcam, UK) at room temperature for 1 h and the blot was developed with a Supersignal chemiluminescence detection kit (Pierce, USA). The primary antibodies used were: rabbit anti-rat polyclonal APN (1:500, Abcam, UK) and rabbit anti-rat polyclonal β-actin (1:2,000, Abcam, UK). The immunoblotting was visualized with a Kodak Image Station 400 (Kodak, USA) and the blot densities were analyzed with Kodak 1D software. Results were expressed as fold change over the control group (right ventricle).

### Statistical analysis

Data were analyzed with SPSS 19.0 software (IBM, US). Results are presented as means±SEM of *n* independent experiments. Analysis of variance (ANOVA) and post hoc analysis were used for comparing means of more than two samples, and Student's *t-*test was used to compare two independent sample means. The correlation analysis was carried out using linear correlation analysis. Values of *P* ≤ 0.05 were considered statistically significant.

## Results

### Establishment of T2DM rat model

In this study, T2DM rat model was established using long-term feeding (32 weeks) with a high-sugar–high-fat diet. Rats fed with the high-sugar–high-fat diet showed listlessness, tiredness, fat body and slow response, while rats fed with normal diet showed full spirit, normal eating and drinking, normal urine color and volume, and free and flexible movement.

**Figure 1:**
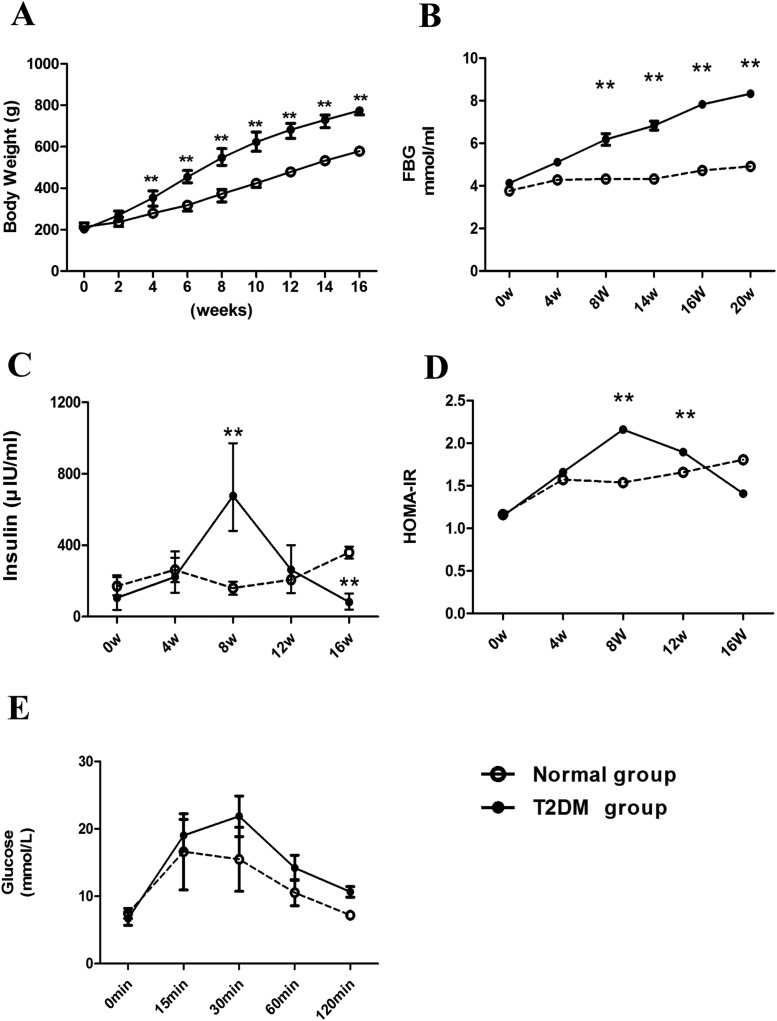
Establishment of type 2 diabetic rat modelwas induced by continuous high-sugar–high-fat feeding. (**A**) Body weight, (**B**) fasting plasma glucose (FPG), (**C**) fasting plasma insulin, (**D**) HOMA-IR and (**E**) intraperitoneal injections of glucose tolerance test (IPGTT) in high-fat–high-glucose diet-induced T2DM model in rats. **P<0.01 *vs*. normal diet group, *n*=23 rats/group.

Compared with the normal rats of the same batch, high-sugar–high-fat rats got a rapidly body weight increase at week 4 (Figure 1A) followed by further continuous and significant increases after that. These rats also had a significant increased fasting blood glucose at week 8 (Figure 1B) followed by continuous increase with the prolongation of feeding with high-sugar–high-fat diet.

There was no significant difference of fasting serum insulin level at week 4 was between normal rats and high-sugar–high-fat rats. However, it increased significantly at week 8 (Figure 1C), and decreased significantly at week 16 (Figure 1C), which suggests that insulin resistance phenomenon occurred at week 8 but insulin deficiency at week 16 in the high-sugar–high-fat diet group.

The HOMA-IR index showed an increased trend in high-sugar–high-fat rats since week 4 and reached the peak value at week 8 (Figure 1D), then gradually dropped, but still significantly higher than that of normal group at week 12. It decreased significantly and was close to the variation trend of fasting insulin at week 16, suggesting a good connection between fasting insulin and HOMA-IR during the establishment of T2DM rat model.

After week 8, the rats on the high-sugar–high-fat diet with a HOMA-IR index significantly higher than that of normal rats of the same batch were selected for IPGTT, once every 4 weeks.

The rats showing a positive result (glucose ≥10 mmol/l 120 min after intraperitoneal injection of glucose, Figure 1E) were labeled asT2DM-positive rats and enrolled into T2DM group. The other rats not meeting the criterion were removed from model group. A total of 23 rats of high-sugar–high-fat diet group were successfully established to the T2DM model at the end of 32 weeks.

### Serum APN level decreases in T2DM rats and is positively correlated with the endothelium-dependent dilation of thoracic aortas

**Figure 2:**
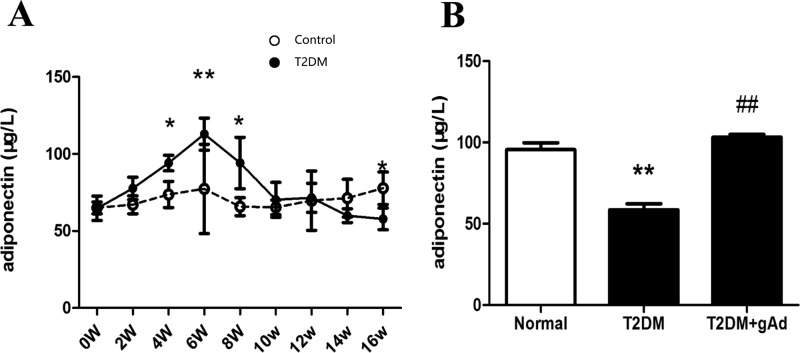
Serum APN level in T2DM rats. (**A**) Dynamic change of APN in high-fat–high-glucose diet-induced T2DM model in rats (*n*=23 rats/group). (**B**) APN level in different groups at the end of 32 weeks (*n*=11–12 rats/group). *P<0.05, **P<0.01 *vs*. normal diet group; **^##^***P*<0.01 *vs*. T2DM group.

During the establishment of T2DM rat models, compared with the normal rats of the same batch, high-sugar–high-fat rats displayed significantly increased APN levels at week 6. Then it decreased, but was still higher than that of normal rats at week 8. At week 12, it gradually decreased, until week 16, when it was significantly lower than that of normal rats (Figure 2A). The low serum APN level lasted until the end of 32 weeks when models were successfully established. It suggested that serum APN level tended to increase following the decrease of insulin and developed an APN resistance phenomenon with a slightly earlier occurrence than insulin at weeks 6–8 during the establishment of T2DM rat models. At the end of 32 weeks, the serum APN level of type 2 diabetic rats was significantly lower than that of that of normal rats (Figure 2B). However, the serum APN level of T2DM rat models significantly rose back after intervention of gAd at the end of 31 weeks (Figure 2B).

**Figure 3:**
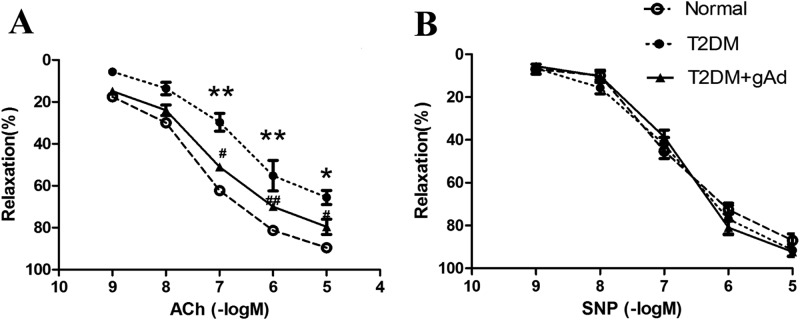
Endothelium-dependent vasorelaxation function in thoracic aorta. (**A**) ACh-induced endothelium-dependent vasorelaxation of thoracic aorta in three experimental groups (*n*=5 rats/group). (**B**) SNP-induced endothelium-independent vasorelaxation remained constant in three experimental groups (*n*=5 rats/group). *P<0.05, **P<0.01 *vs.* normal diet control group; ^#^*P* <0.05, ^##^*P*<0.01 *vs.* T2DM group.

The thoracic aortas of T2DM rat models were collected at the end of 32 weeks. The endothelium-dependent and -independent diastolic functions were detected with acetylcholine (ACh) and sodium nitroprusside (SNP), respectively. As the results showed, ACh-induced endothelium-dependent diastolic function of the thoracic aorta had a maximum diastolic value of 70% in T2DM rats, which was significantly lower than that in normal rats (with a maximum diastolic value of 93%), but it was significantly improved with a maximum diastolic value of 81% after incubating the thoracic aorta rings of T2DM rats with gAd (2 μg/ml) for 4 h (Figure 3A). However, there was no significant difference of SNP-induced endothelium-independent diastolic function between the two groups (Figure 3B). All these result suggest that long-term stimulation of high-sugar–high-fat induced vascular endothelial dysfunction, and the appropriately increasing APN level alleviated the endothelium dependent diastolic dysfunction.

**Figure 4:**
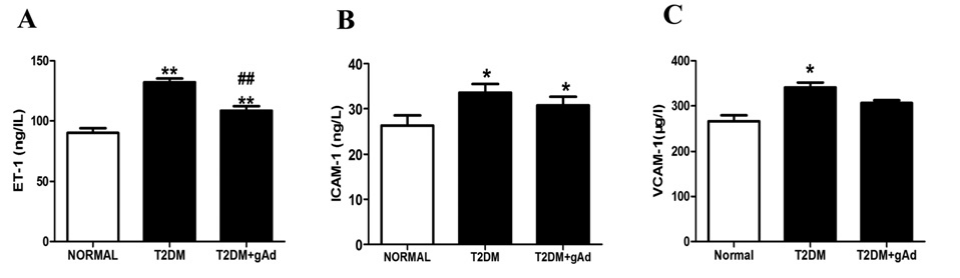
Serum ET-1, ICAM-1 and VCAM-1 were increased in type-2 diabetic rats, and gAd treatment reversed high-sugar–high-fat diet-related upregulation of serum ET-1 and ICAM-1. (**A**) Serum ET-1, (**B**) ICAM-1 and (**C**) VCAM-1 in T2DM rats at the end of 32 weeks. *P< 0.05, **P< 0.01 *vs*. normal diet group; **^##^***P*<0.01 *vs*. T2DM group. *n*=11–12 rats/group.

Meanwhile, the serum endothelin-1 (ET-1) level was increased significantly in T2DM rat models compared with that in normal (Figure 4A), suggesting that long-term toxicity of high sugar–high fat had caused damage to the endothelium of DM rats, which may be one of the important reasons for the dysfunction of vascular endothelium. A trend of damage alleviation appeared after administration of exogenous gAd (Figure 4A). In addition, serum ICAM-1 (Figure 4B) and VCAM-1 (Figure 4C) contents were significantly increased in T2DM rat models, suggesting that the myocardial microcirculation system of T2DM rats might be damaged. Serum ICAM-1 content was slightly decreased after administration of exogenous gAd.

**Figure 5:**
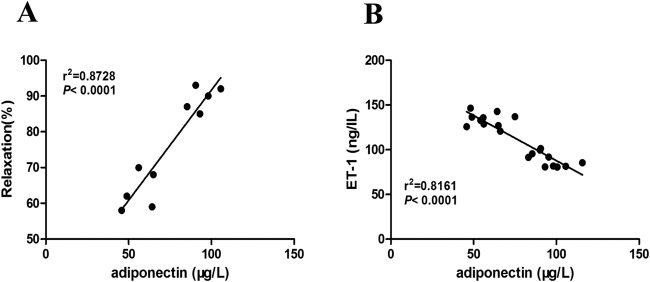
Correlation analysis of serum APN level with endothelium-dependent vasorelaxation function of thoracic aorta and serum ET-1 level. (**A**) Positive correlation between serum APN level and endothelium-dependent vasorelaxation function of thoracic aorta in T2DM rats at the end of 32 weeks (*n*=10). (**B**) Negative correlation between serum APN level and serum ET-1 level in T2DM rats at the end of 32 weeks (*n*=20).

In order to further confirm the relationship between the change of serum APN level and vascular endothelial dysfunction and damage in T2DM rats, a correlation analysis was performed. It was found that serum APN level at the end of 32 weeks was positively correlated with the endothelium-dependent diastolic function of the thoracic aortic (Figure 5A), but negatively correlated with serum ET-1 (Figure 5B). It was confirmed that vascular endothelial dysfunction and damage had a linear correlation with serum APN level in T2DM rats.

### Myocardial NR injury aggravates after MI/R and expression of APN protein decreases in NR area of T2DM rats

**Figure 6:**
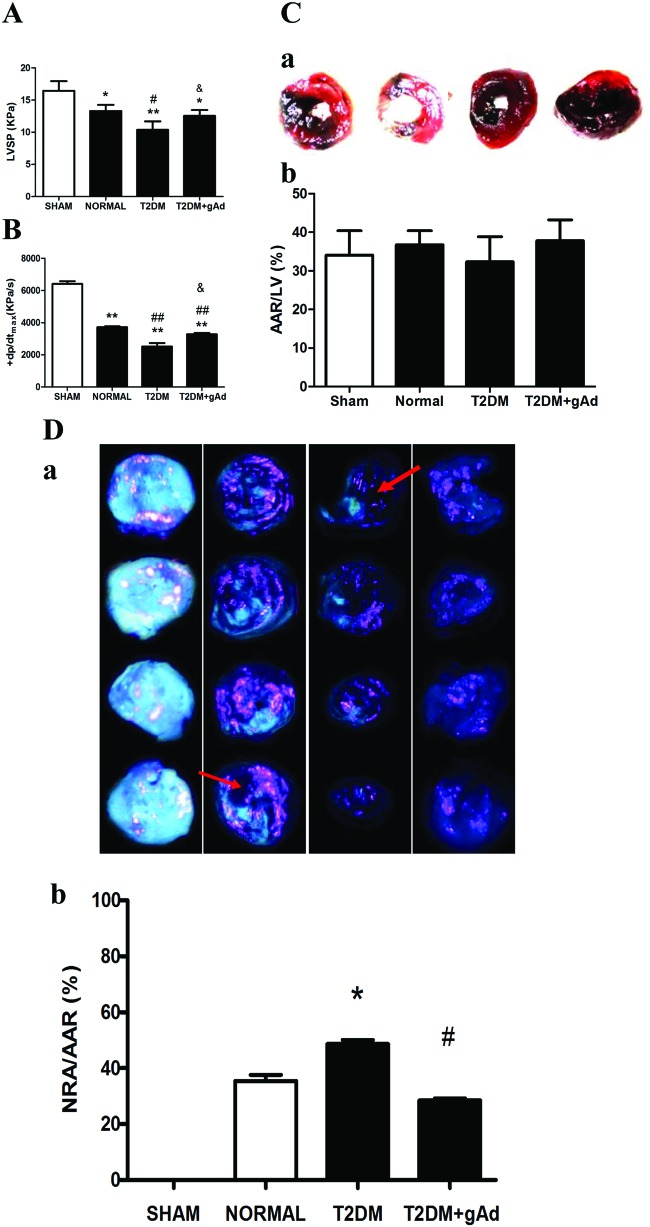
NR injury in T2DM rats was increased after MI/R, and gAd treatment ameliorated the NR injury. (**A**) LVSP, (**B**) +dp/dt_max_, (**C**) AAR/LV and (**D**) NRA/AAR in different treatment groups subjected to MI/R. (**C-a**) and (**D-a**), representative photographs of heart sections obtained from rats in sham, normal diet, DM with vehicle and gAd groups subjected to MI/R. (**C-a**) Dark portion by Evan's blue staining: non-ischemic, normal region; red portion: ischemic area (area at risk, AAR), negatively stained by blue dye. (C-b) Myocardial ischemic area (AAR) in different groups. (**D-a**) Bright blue-green fluorescence portion by Thioflavin-S staining: reflow area; bright pink fluorescence portion by fluorescent microsphere staining: reflow area; negatively stained by fluorescence: no reflow area. (**D-b**) No reflow area expressed as percent of AAR. *P<0.05, **P<0.01 *vs*. sham groups; ^#^*P*<0.05, ^##^*P*<0.01 *vs*. normal diet group; ^&^*P* <0.05 *vs*. T2DM group. *n*=6–7 rats/group.

The coronary NR rat model was established successfully by MI/R operation. LVSP (Figure 6A) and +dp/dt_max_ (Figure 6B) were clearly decreased after MI/R in T2DM rats compared with that in normal diet rats, which meant that myocardial systolic dysfunction aggravated after MI/R in T2DM rat models.

After MI/R, the influence of surgical techniques on experimental results was excluded according to the ratio of ischemia area to left ventricular area (there was no statistical difference in four groups, Figure 6C). NR area was determined based on Thioflavin S, Evan's blue fluorescent microsphere staining after MI/R. The staining result showed that NR area was increased significantly (Figure 6D) in T2DM rats compared with that in normal diet rats, suggesting that the long-term stimulation with high-sugar–high-fat might cause more serious coronary NR after MI/R.

**Figure 7:**
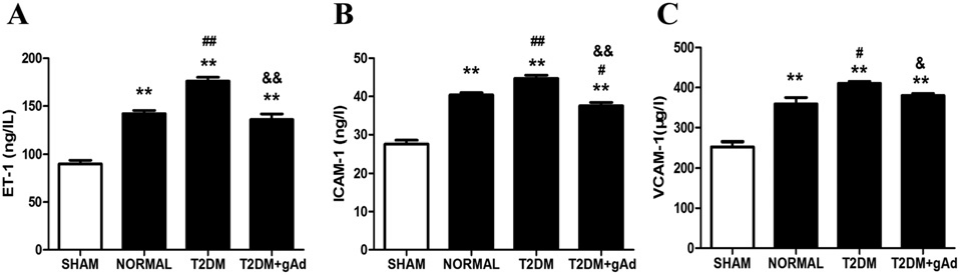
Serum ET-1, ICAM-1 and VCAM-1 were significantly increased in the DM group after MI/R, and gAd treatment improved endothelium injury and microcirculation disturbance. (**A**) Serum ET-1, (**B**) ICAM-1 and (**C**) VCAM-1 in different treatments groups subjected to MI/R. **P<0.01 *vs*. sham groups; **#***P*<0.05, **^##^***P*<0.01 *vs*. normal diet group; **^&^***P*<0.05, **^&&^***P*<0.01 *vs*. T2DM group. *n*=6–7 rats/group.

The level of serum ET-1, ICAM-1 and VCAM-1 were also increased significantly (Figure 7A-C) after the MI/R surgery compared with normal diet rats, which suggested that vascular endothelial injury and the dysfunction of myocardial microcirculation were aggravated further in T2DM rats after MI/R, which may be one of the reasons for the aggravation of NR injury.

**Figure 8:**
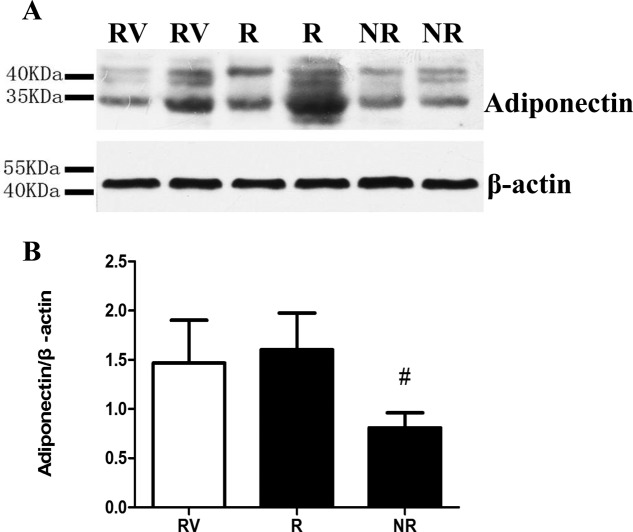
APN protein expression in the ischemia area of type 2 diabetic rats. **^#^***P<*0.05 *vs*. area of no reflow. *n*=6–7 rats/group. RV: right ventricular; NR: no reflow; R: reflow.

The expression of APN protein in the myocardial tissues of the right ventricle, NR and reflow areas in the same rat were determined respectively, after Thioflavin S and Evan's blue fluorescent microspheres staining. The expression of myocardium APN was obviously lower in NR area than that in reflow zone (Figure 8), suggesting that APN level might be the fundamental reason for the aggravation of NR injury in T2DM rats, and that endothelium injury and myocardial microcirculation dysfunction might influence the biological functions of APN.

### Coronary NR injury after MI/R was alleviated by supplementing exogenous APN

LVSP (Figure 6A) and +dp/dt_max_ (Figure 6B) rose significantly after MI/R and the function of myocardial contraction was dramatically improved in T2DM rats after supplementing gAd. Meanwhile, the area of myocardial NR significantly shrank (Figure 6D) and the levels of serum ET-1, ICAM-1 and VCAM-1 were decreased significantly (Figure 7A-C).

## Discussion

In this study, SD rats were fed with high-sugar and high-fat food for 32 weeks to build T2DM rat models and were given with the intervention of exogenous gAd at the end of 31 weeks. The results of our study showed that serum APN level in T2DM rat models was decreased at the end of 32 weeks and the variation trend of serum APN level was close to that of serum insulin. An APN resistance phenomenon had an slightly earlier occurrence than that of insulin. Endothelium-dependent diastolic function in the thoracic aorta of T2DM rat models was decreased, and the levels of serum ET-1 level and adhesion molecules (ICAM-1 and VCAM-1) were increased. After intervention with exogenous gAd, the function of endothelium-dependent vasodilatation was improved in some degree and the level of serum ET-1 was decreased. Additionally, the level of serum APN showed respectivel positive and negative correlation with the maximum diastolic function of endothelium-dependent vasodilatation in the thoracic aorta and the level of serum ET-1. The cardiac function of T2DM rat models was impaired significantly after ischemia/reperfusion. The NR area was increased, while the expression level of myocardium APN protein in NR area was noticably decreased. The level of serum ET-1 and adhesion molecules (ICAM-1 and VCAM-1) were distinctly increased. Meanwhile, the cardiac function was notably improved, the NR area was decreased, and the level of serum ET-1 and adhesion molecules (ICAM-1 and VCAM-1) were also reduced. The results of our study suggested that APN can reduce effectively the injury of coronary NR after T2DM rat models suffered with MI/R, which may be realized by the protection of endothelial tissue and improvement of microcirculation.

Cardiovascular disease is one of major complication in diabetes, accounting for more than 50% of the fatality rate [12]. The morbidity and mortality of ischemic heart disease in diabetic patients are considerably higher than the normal population. Myocardial ischemic areas of these patients are serious and the incidence of heart failure is obviously increased with post-myocardial infarction [13]. The determination results of cardiac function and NR area were consistent with related reports in this study, suggesting that the occurrence risk of coronary NR was raised after T2DM was treated with reperfusion. It was reported that ischemic injury of endothelial cells was along with microvascular obstruction, leukocyte and platelet effect, oxygen free radical mediated microvascular injury, vascular dysfunction, tissue factor and coagulation effect, micro-embolization, mechanical nerve compression and so on, but the underlying mechanisms of this phenomenon are not completely understood yet. Moreover, the patients with NR may have individual differences, thus the mechanism of NR needs to be studied further in order to establish personalized medicine, which is key to improving therapeutic efficacy.

Diabetic microangiopathy disease is found mainly in the retina, kidney, myocardium, nerve tissue and toe. In this study, we focused on the influence of diabetic microangiopathy on macrovascular disease. In other words, the dysfunction of myocardial microcirculation affected the coronary NR in T2DM after MI/R. Macrovascular and microvascular disease have common features with regard to endothelial dysfunction in T2DM [13], including damage of vascular endothelial cells, dysfunction of endothelium-dependent vasodilation, disorder of hemodynamics, reduction of fibrinolytic ability, production of numerous growth factors, overexpression of adhesion molecules and inflammatory cytokine, excess of ROS generatation, increase of oxidative stress and cell permeability [14–18].

In the progression of severe angina, AMI and MI/R injury, the synthesis and release of ET-1 are increased significantly and the vascular affects ET-1 with hyperactivity. Additionally, the level of serum ET-1 in patients with ACS has positive correlation with the occurrence and severity of coronary NR [19,20]. Previous research has shown that NR is another manifestation of progressive myocardial damage and microcirculation injury after AMI was recovered with reperfusion. The system of microcirculation injury is the main battlefield where NR occurs. ICAM-1 and VCAM-1 belong to the immunoglobulin of superfamily molecule, and regulate cell–cell and cell–extracellular matrix to contract and combine with each other. Overexpression of ICAM-1 leads to increased endothelial cell permeability, which facilitates leukocyte deformability enabling them to pass through endothelial cells and basement membrane to gather and infiltrate in local tissue, further causing myocardial microvascular dysfunction and myocardial tissue damage [21,22]. In the present study, the function of endothelium-dependent vasodilation in the aorta was observed in T2DM rats. Meanwhile, the level of serum ET-1, ICAM-1 and VCAM-1 was further increased after ischemia/reperfusion. This further revealed that the occurrence of coronary NR was closely related with vascular endothelial injury and myocardial microcirculation in T2DM with acute MI/R.

APN is primarily synthesized and secreted by fat cells, which is a highly negative correlation with varieties of metabolic diseases, such as, obesity, hypertension, high cholesterol and T2DM [23]. A large number of clinical trials have shown that populations with higher APN levels have a lower chance of diabetes and are lesslikely to suffer from cardiovascular disease [24]. Animal experiments show that insulin resistance is improved noticably with exogenous APN. The myocardial ischemic area of APN-knockout mice is larger than that of normal mice after ischemia/reperfusion. While the myocardial ischemic area reduces with the treatment of exogenous APN [25]. These results imply that APN is not only related with the occurrence of diabetes, but also may be one of key factors that influence on the occurrence of ischemic heart disease and prognosis. In this study, the decrease of serum APN was correlated with the increase of the area of coronary NR after I/R and the decreased heart function in T2DM rats. While NR was improved, perfusion was increased and heart function was improved after these rats were given exogenous APN, suggesting that the reduced expression level of APN may be one of factors leading to an increase of NR in T2DM. In addition, we examined protein expression of APN in NR and recovery perfusion in ischemic heart tissue in T2DM rats, and results revealed the APN protein expression in the former was less than that of the latter, implying that microcirculation dysfunction may affect APN levels and further affect its biological function.

APN inhibits monocyte adhesion to endothelial cells by reducing the delivery of intracellular NF-κB (nuclear factor κB) and the expression of adhesion molecules on vascular endothelial cells, resulting inan anti-atherosclerotic effect [26]. Kusmic et al. [27] found that APN improved myocardial perfusion by increasing the signal of pLKB1 and AMPK (AMP-activated protein kinase). Zhao et al. [28] demonstrated that APN improved skeletal muscle microcirculation. Alvarez et al. [29] found that APN promoted the proliferation of human endothelial cells. APN also inhibited the output of inflammatory cytokines and adhesion molecules, including intercellular adhesion molecule, vascular cell adhesion molecules, E-selectin on endothelial cells [30]. Moreover, APN repaired vascular damage caused by angiotensin II by mediating the activation of AMPK [31]. Our previous study also found that APN prevented endothelial injury in various ways, such as C1q/TNF-related protein and APN receptors-1/AMPK/eNOS/nitric oxide signal pathways, and further induced vasodilation [32]. In addition, APN inhibited oxidative stress and reduced the vascular damage caused by itself through protein kinase A-dependent NF-κB signal pathways with I/R [33]. At the same time, it inhibited vascular inflammation caused by tumor necrosis factor α (TNF-α) through caveolin-mediated neuraminidase accumulation and activation [34]. These results suggested that high APN levels protected vascular endothelial cells, and improved endothelium-dependent vasodilation and microcirculation. The results of this study showed that after incubation of APN, endothelium-dependent vasorelaxation in the aorta was improved; serum ET-1 and the elevated levels of ICAM-1 and VCAM-1 were obviously reduced after T2DM rats with MI/R, suggesting that APN played a role in relieving coronary NR effect by protecting endothelial cells and improving the function of microcirculation.

In conclusion, our study confirmed that hypoadiponectinemia was related to the aggravated degree of coronary NR in T2DM rats, and APN protects endothelial cells and improves myocardial microcirculation to alleviate the coronary NR injury in T2DM rats. This results suggest that APN is related to ischemic heart disease that caused by multiple risk factors, that is to say, APN in the treatment of cardiovascular disease may have broad applications in addition to regulating glucose and lipid metabolism disorders, which provide a new target and theoretical basis for the prevention of coronary NR in clinical practice and decreasing MI/R injury.

Due to the limitation of diagnostic techniques, current basic experiments of NR mainly used large animals, for example, pigs, dogs or rabbits. However, the application of these large animals to build a T2DM model suffers from multiple restrictions, such as costs and technology. Thus, our group built a T2DM model by feeding rats with long-term high-sugar and -fat food. Based on this, the surgery of I/R was carried out, and the success of this model was identified by Thioflavin-S–Evan's blue-fluorescent microsphere staining. However, we are still unable to apply a classical clinical diagnosis of coronary NR via angiography due to the limitations of the rat body. In this study, Thioflavin-S–Evan's blue-fluorescent microsphere staining was just used for model identification. Nevertheless, two kinds of diagnostic techniques, including delayed enhancement of MRI and radionuclide imaging, were actively explored in order to further improve the establishment of coronary NR rat model.

## Abbreviations

ACS: acute coronary syndrome
AMI: acute myocardial infarction
AMPK: AMP-activated protein kinase
APN: adiponectin
DM: diabetes mellitus
FBG: fasting blood glucose
FINS: fasting serum insulin
gAd: globular domain of adiponectin
HOMA-IR: homeostasis model assessment of insulin resistance
IPGTT: intraperitoneal injections of glucose tolerance test
LAD: left anterior descending
LV: left ventricular
LVEDP: left ventricular end diastolic pressure
LVSP: left ventricular systolic pressure
MI/R: myocardial ischemia/reperfusion
NF-κB: nuclear factor κB
NR: no-reflow
PCI: percutaneous coronary intervention
SD: Sprague–Dawley
T2DM: type 2 diabetes mellitus
